# Radiation of nitrogen‐metabolizing enzymes across the tree of life tracks environmental transitions in Earth history

**DOI:** 10.1111/gbi.12419

**Published:** 2020-10-27

**Authors:** Chris Parsons, Eva E. Stüeken, Caleb J. Rosen, Katherine Mateos, Rika E. Anderson

**Affiliations:** ^1^ Carleton College Northfield MN USA; ^2^ Massachusetts Institute of Technology Cambridge MA USA; ^3^ University of St Andrews, St. Andrews Scotland, UK; ^4^ NASA NExSS Virtual Planetary Laboratory University of Washington Seattle WA USA

**Keywords:** denitrification, horizontal gene transfer, microbial evolution, nitrogen cycle, nitrogenase

## Abstract

Nitrogen is an essential element to life and exerts a strong control on global biological productivity. The rise and spread of nitrogen‐utilizing microbial metabolisms profoundly shaped the biosphere on the early Earth. Here, we reconciled gene and species trees to identify birth and horizontal gene transfer events for key nitrogen‐cycling genes, dated with a time‐calibrated tree of life, in order to examine the timing of the proliferation of these metabolisms across the tree of life. Our results provide new insights into the evolution of the early nitrogen cycle that expand on geochemical reconstructions. We observed widespread horizontal gene transfer of molybdenum‐based nitrogenase back to the Archean, minor horizontal transfer of genes for nitrate reduction in the Archean, and an increase in the proliferation of genes metabolizing nitrite around the time of the Mesoproterozoic (~1.5 Ga). The latter coincides with recent geochemical evidence for a mid‐Proterozoic rise in oxygen levels. Geochemical evidence of biological nitrate utilization in the Archean and early Proterozoic may reflect at least some contribution of dissimilatory nitrate reduction to ammonium (DNRA) rather than pure denitrification to N_2_. Our results thus help unravel the relative dominance of two metabolic pathways that are not distinguishable with current geochemical tools. Overall, our findings thus provide novel constraints for understanding the evolution of the nitrogen cycle over time and provide insights into the bioavailability of various nitrogen sources in the early Earth with possible implications for the emergence of eukaryotic life.

## 1 INTRODUCTION

**Figure 1:**
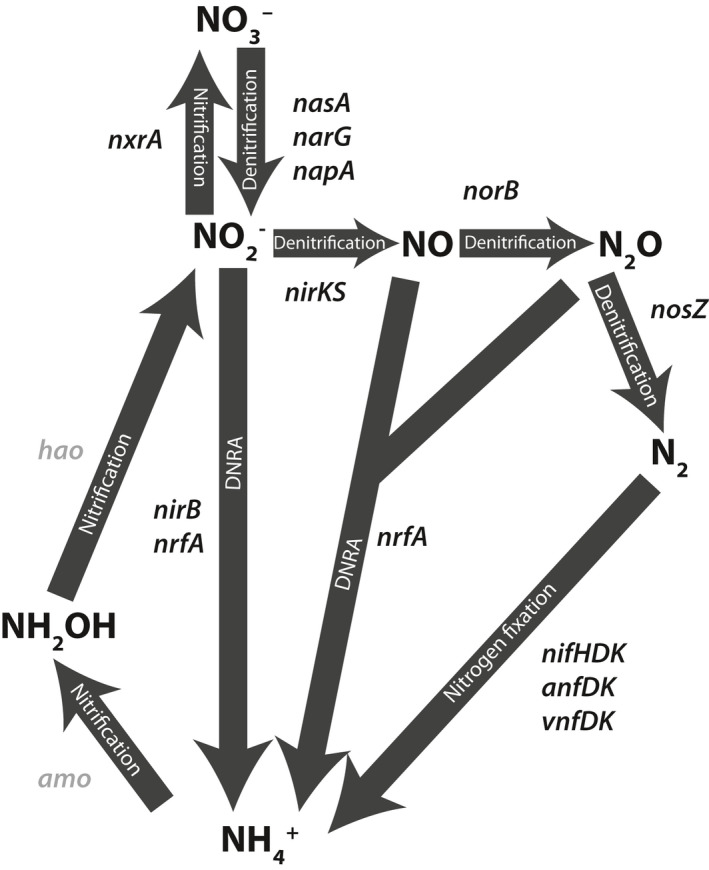
Schematic of the biological nitrogen cycle. Arrows are labeled with the pathway to which they belong. All genes examined in this study are labeled next to the step they catalyze. Genes without sufficient data for subsequent analysis are labeled in gray. Adapted from Canfield et al. (2010)

Nitrogen is a critical element to life on Earth, important as an essential building block in the synthesis of biological molecules, and for its role in redox reactions for microbial energy metabolism. It is often a limiting nutrient in marine and terrestrial environments and likely had a significant influence on the evolutionary trajectory of the biosphere over Earth's history. The nitrogen cycle is largely controlled by a variety of microorganisms that enzymatically catalyze the reduction and oxidation of nitrogen at various redox states (Figure 1). Reconstructing the genetic proliferation of these enzymes across the tree of life through gene birth, duplication, loss, and horizontal gene transfer can therefore provide novel insights into the evolution of the biosphere and its productivity over time.

The most important steps in Earth's nitrogen cycle are largely catalyzed by microbes, including the first crucial step of reducing molecular nitrogen to bioavailable forms (Kuypers et al., 2018; Zerkle & Mikhail, 2017). Nitrogen fixation is catalyzed by nitrogenase, of which there are three varieties, distinguished by the metals in their associated active site cofactors: Nif (Fe‐Mo), Vnf (Fe‐V), and Anf (Fe‐Fe) (Joerger et al., 1988; Miller & Eady, 1988) (Figure 1). The ability to fix nitrogen is spread across a wide range of archaeal and bacterial lineages, but does not occur in eukaryotes (Dos Santos et al., 2012; Gaby & Buckley, 2014). Importantly, nitrogenase is strongly inhibited by oxygen, forcing nitrogen fixers to develop various means to reduce their intracellular oxygen concentrations or to confine themselves to suboxic environments (Gallon, 1981). Ammonium produced from nitrogen fixation or ammonification is converted to organic forms of fixed nitrogen by a variety of enzymes or, in the presence of oxygen, oxidized to nitrite (NO_2_^‐^) or nitrate (NO_3_^‐^) through the chemoautotrophic nitrification pathway via the enzymes Amo and Hao (Figure 1). In environments with insufficient O_2_ concentrations to support aerobic respiration, nitrate and nitrite can be utilized as alternative terminal electron acceptors through the denitrification pathway; consequently, denitrification rates are highest in suboxic conditions, including swamps and marine oxygen minimum zones (Canfield et al., 2005; Löscher et al., 2012; Voss et al., 2013). Reduction of nitrate to nitrite, nitric oxide (NO), nitrous oxide (N_2_O), and dinitrogen (N_2_) is catalyzed by the Nas, Nar, Nap, Nir, Nor, and Nos enzymes, respectively. Dissimilatory nitrate reduction to ammonium (DNRA) also reduces nitrate via the enzymes Nar, Nap, Nir, and Nrf (Figure 1), but this pathway retains fixed nitrogen as ammonium and may therefore have been a critical metabolism in nutrient‐starved ecosystems. DNRA, while less understood than denitrification, has been shown to be a major nitrate sink in a variety of aquatic systems, especially warm intertidal zones (Giblin et al., 2013) and it may be dominant under ferruginous conditions, as suggested by modern analogue studies (Michiels et al., 2017).

Given the importance of nitrogen as a building block of life, as an energy source for microbes, and as the most abundant element in the Earth's atmosphere, better constraining the evolutionary history of the nitrogen cycle is important for understanding, among other things, variation in global primary productivity and atmospheric pressure over time. The bioavailability and cycling of important limiting nutrients through Earth's history, including nitrogen, would have been important for biological productivity and the rise of early eukaryotic algae (Anbar & Knoll, 2002; Isson et al., 2018; Sánchez‐Baracaldo et al., 2014). The relative abundance of nitrogenous gases in the atmosphere could also have had important implications for atmospheric pressure as well as planetary climate during the Archean. Potential changes in atmospheric pressure during the Archean may have resulted from biological N_2_ drawdown (Som et al., 2016), whereas the greenhouse gas nitrous oxide (N_2_O), produced as part of the nitrogen cycle via nitrification/denitrification, may have contributed to planetary warming when the Sun was younger and fainter (Buick, 2007; Roberson et al., 2011; Stanton et al., 2018). Finally, one of the most important questions for the early evolution of life is understanding when fixed nitrogen first became widely available, and through what means. Experimental data suggest that fixed nitrogen can be produced during lightning reactions and under hydrothermal conditions (e.g., Brandes et al., 1998; Navarro‐González et al., 2001), and either or both of these sources were likely pivotal for the origin of life. However, the invention of biological N_2_ fixation would have made Earth's biosphere less dependent on abiotic reactions and likely spurred primary productivity.

The question of how these metabolisms unfolded over Earth's history has previously been addressed with both geochemical and phylogenetics‐based approaches. Geochemical approaches, relying on the reconstruction of metabolisms based on the rock record, have suggested that biological nitrogen fixation emerged early (Koehler et al., 2019; Ossa Ossa et al., 2019; Stüeken et al., 2016) and that the nitrogen cycle expanded considerably during the Neoarchean (2.8–2.5 Ga) and Paleoproterozoic (2.5–1.8 Ga) (Garvin et al., 2009a; Godfrey & Falkowski, 2009; Kipp et al., 2018; Koehler et al., 2018; Luo et al., 2018; Zerkle, Poulton, et al., 2017). Phylogenetics studies, relying on sequence data, reconstruct the evolutionary history of genes of interest (e.g., Boyd et al., 2011; Garcia et al., 2020; Jones et al., 2008), and some molecular clock studies have yielded conservative estimates for the approximate timing of an enzyme's origin (Boyd, et al., 2011; Boyd & Peters, 2013; Raymond et al., 2004). While each approach provides valuable insights, they both have weaknesses. Geochemical data cannot reliably distinguish between all enzymatic pathways, because the isotopic effects of some reactions (e.g., denitrification, DNRA, and ANAMMOX) are too similar to each other. Furthermore, geochemical data, which are typically collected from bulk rock samples, only preserve evidence of the most dominant metabolisms and may therefore not capture the origin of new enzymes until they gain ecological significance. Conversely, the phylogenetic approach of dating the antiquity of enzymes does not show when these enzymes gained ecological dominance. Furthermore, most phylogenetic studies have so far focused on nitrogenase, leaving the evolutionary history of most nitrogen‐cycling enzymes poorly constrained. Additional work is therefore needed to address key questions about the dynamics of the nitrogen cycle on the early Earth and its evolution over time. The proliferation of sequencing data over the past decade has made vast amounts of genomic data available, which can provide novel insights into the evolution of nitrogen‐cycling genes over time.

As a new approach to these questions, we track the timing of birth, speciation, duplication, loss, and horizontal gene transfer events for genes involved in each step of the nitrogen cycle, which can provide contextual information for the rise and spread of key nitrogen‐cycling genes across the tree of life. We place particular focus on the acquisition of new genes via horizontal gene transfer (HGT), which is common in microbial lineages and is a crucial evolutionary mechanism by which a clade of organisms can develop new and useful phenotypes without the evolutionary cost associated with independently evolving genes (Beiko et al., 2005; Gogarten & Townsend, 2005). Studies of gene gain and loss have revealed a history of widespread HGT throughout the microbial tree of life, which we have attempted to leverage as a means to attribute trends in microbial evolution to events in Earth history (Koonin et al., 2001; Mirkin et al., 2003). Many of the major genes in the nitrogen cycle have been shown to have experienced extensive HGT, presumably due to their modularity and their strong dependence on oxygen availability (Jones et al., 2008; Kechris et al., 2006; Stolz & Basu, 2002). We tracked birth, speciation, duplication, loss, and HGT of genes in the nitrogen cycle over time by comparing the phylogenies for specific nitrogen‐metabolizing genes to a time‐calibrated tree of life to demonstrate when these genes first arose and then spread across the tree of life on the early Earth.

## 2 MATERIALS AND METHODS

### 2.1 Genome selection and compilation

The construction of both the gene and species trees for this study was based upon the manual curation of a genome database containing 308 genomes (including 254 bacterial and archaeal genomes) that served as the basis for the species tree and was subsequently searched to find genes related to nitrogen metabolism. Assembled genomes were downloaded from ggKBase (Hug et al., 2016) and the NCBI assembly database (Kitts et al., 2016). Additionally, six genomes were collected from a recent study identifying novel nitrogen fixers (Delmont et al., 2018). In constructing the tree, we included at least one genome from each bacterial or archaeal phylum represented in the most recent comprehensive tree of life (Hug et al., 2016) in order to create a tree fully representative of our current understanding of microbial diversity. It also includes a set of genomes associated with a database of *nifH* genes (Gaby & Buckley, 2014). Some eukaryotic genomes were included for construction of the species tree, but for this study we focused only on archaeal and bacterial genomes for identification of nitrogen‐cycling genes. Relative to archaea and bacteria, eukaryotes play a more minor role in the nitrogen cycle—while some species of fungi and protists reduce nitrate or nitrite to more reduced forms of nitrogen, there are no known eukaryotes that mediate nitrogen fixation, nitrification, DNRA, or anammox (Stein & Klotz, 2016).

### 2.2 Species tree and chronogram construction

To create the species tree, all bacterial and archaeal genomes in our database were mined for single‐copy ribosomal protein sequences L2, L3, L4, L5, L6, L14, L15, L16, L18, L22, L24, S3, S8, S10, S17, and S19 using Phylosift (Darling et al., 2014), with the isolate and best hit command line flags. These 16 ribosomal proteins represent the same proteins used to create a recent comprehensive tree of life (Hug et al., 2016) and, in the case of eukaryotes, ribosomal sequences were directly drawn from their dataset. All genomes included in the dataset contained fewer than 50% gaps in the alignment. These 16 single‐copy ribosomal proteins were concatenated to create a final alignment of 2,897 characters for phylogenetic reconstruction and molecular clock evaluation.

Alignments for the species trees were made using the Phylosift pipeline (Darling et al., 2014) and curated to only include the target ribosomal proteins listed above. The phylogeny was constructed using RAxML v.8.2.9 with 100 rapid bootstraps (Stamatakis, 2014). The CAT model, which calculates site‐specific evolutionary rates, was used with an LG substitution matrix to construct the species tree based on reference marker genes (the species tree). The root for the species tree was placed in the Bacterial domain (Fournier & Gogarten, 2010).

### 2.3 Chronogram construction

The species tree was converted into chronograms using PhyloBayes (Lartillot et al., 2009) using different clock models and calibration points in order to test the sensitivity of our results to variation in Phylobayes parameters. The root age was set via a normally distributed gamma root prior according to the liberal or conservative calibration points set in Table 1 and Table S1, with standard deviation set to 200 in accordance with previous studies (Magnabosco et al., 2018). We tested two separate sets of calibration points, one liberal (which represents the earliest date for which there is any evidence of a given event based on the current scientific literature) and one conservative (which represents the earliest date for which there is the most consensus for a given event based on the current scientific literature), to test the sensitivity of the methodology (Table 1 and Table S1). For each set of internal calibration points, the ages in the calibration files were set as the hard lower bound for the analysis. The liberal calibration points shown in Table S1 yielded unrealistic root ages (>4.5 Ga) and therefore were not used for further analyses.

**Table 1:** Fossil calibration points used in Phylobayes runs. Calibration points were set as the hard constraint indicating the latest date by which a specific clade split. The selected time points reflect the dates for which there is the most consensus

Calibration event	Date (Mya)	Refs
LUCA (set as root prior)	3,800, 200 S.D.	Schidlowski et al. (1983); Schidlowski (1988); Mojzsis et al. (1996); Rosing (1999); Czaja et al. (2013); Nutman et al. (2016)
Origin of Methanogenesis	>2,700	Eigenbrode and Freeman (2006)
Origin of Cyanobacteria	>2,450	Bekker et al. (2004)
Origin of Eukaryotes	>1,700	Pang et al. (2013)
Origin of plastids/Rhodophytes diverge	>1,050	Gibson et al. (2018)
Akinetes diverge from cyanobacteria lacking cell differentiation	>1,000	Pang et al. (2018)

We created chronograms using both the uncorrelated gamma (UGAM) model (Drummond et al., 2006) and the autocorrelated CIR relaxed clock model (Lepage et al., 2007) to compare the effects of clock model type on the results. Two chains were run in parallel for each set of parameters, so that the two concurrent runs could be compared to one another as a test of convergence. Convergence of the MCMC chains was checked visually by plotting the summary statistics and quantitatively by comparing the posterior distributions of two parallel chains using the *tracecomp* and *bpcomp* programs in PhyloBayes. We required an effective size >100 and a maximum difference between chains of <0.3. Simultaneous chains were run for approximately 36,000 cycles. Chronograms were generated using the *readdiv* function in Phylobayes 4.1, with approximately 20% of initial cycles discarded as burn‐in. Chronograms were visualized using the phytools package in R (Revell, 2012). In order to test for the influence of the priors, we generated additional chronograms in the absence of sequence data using the *‐* *prior* flag in Phylobayes. These chronograms displayed substantially different node timings, demonstrating that the priors did not overly influence the inferred dates and the sequence data informed the chronogram. Results from molecular clock analyses should be interpreted with caution, given the limitations associated with these analyses, including but not limited to changing generation times, the influence of natural selection, and variation in mutational rates across species (Ayala, 1999; Bromham et al., 2018; Schwartz & Maresca, 2006). Here, we attempted to ameliorate some of these challenges by using methods allowing for variation of rates between and across lineages, and by comparing results produced by different clock models. Finally, our goal with this analysis was not to pinpoint exact dates for many of the transitions discussed here, but rather to compare the relative timing on broad evolutionary scales.

### 2.4 Identification of nitrogen‐cycling genes and construction of gene trees

Curated gene database queries for each of the nitrogen‐cycling genes, we investigated were generated based on KEGG orthologies (Ogata et al., 1999) and downloaded from the UniProt database (The Uniprot Consortium, 2017). Nitrogen‐cycling amino acid sequences for the gene trees were identified by conducting BLASTP (Altschul et al., 1990) searches of the open reading frames (ORFs) of every genome from the collection of 254 genomes. All ORFs were identified using Prodigal (Hyatt et al., 2010). The maximum e‐value cutoff for BLAST hits was 10^–12^ and matches were excluded if the length of the local alignment was <50% of the length of the query sequence. BLAST results were compared with results from AnnoTree (Mendler et al., 2019) to verify gene distributions. Given that *nifK* and *nifD* have a shared evolutionary history (Fani et al., 2000), an e‐value cutoff of 10^‐30^ was selected based on close examination of BLASTP hit results and KEGG annotations, which was sufficient to distinguish these subunits. For nitrogenase subunits *nifH*, *vnfD, vnfK, anfD, and anfK,* we manually curated alignments by identifying key residues that were crucial for enzyme structure and function, as determined through literature searches and visualization using PyMol (Brigle et al., 1987; Howard et al., 2013; Kaiser et al., 2011; Keable et al., 2018; McGlynn et al., 2012). Jalview (Waterhouse et al., 2009) was used for visualization of key residues in alignments. All genes that did not include the key residues were removed from the alignment. Finally, to ensure that only the genes of interest were included in alignments and to verify annotations, all genes identified in the BLAST search were compared to the KEGG database using Kofam Koala (Aramaki et al., 2019), which assigns KEGG Ortholog numbers to each gene by using a homology search against a database of profile hidden Markov models. Only genes verified to be the gene of interest according to Kofam Koala were retained for downstream analysis. It is important to note that gene identification is necessarily limited by the search tools and databases used for annotation, and the methods used here were chosen to be conservative so as to ensure the removal of non‐target genes from the analysis.

Alignments for the gene trees were created using MUSCLE (Edgar, 2004) and trimmed with TrimAl (Capella‐Gutierrez et al., 2009) using the *‐* *automated1* option. The model of evolution was selected using Model Selection as implemented in IQ‐TREE (Kalyaanamoorthy et al., 2017) using the default parameters. Trees were generated with RAxML‐NG (Kozlov et al., 2019) using the model of evolution identified in IQ‐TREE. Trees were run with at least 1,000 bootstraps or until the diagnostic statistic based on the MRE‐based bootstrapping test as implemented in RAxML‐NG dropped below a cutoff of 0.03.

### 2.5 Gene tree and species chronogram reconciliation

Gene trees were reconciled with species chronograms using the Analyzer of Gene and Species Trees (AnGST) (David & Alm, 2011). AnGST compares the topology of the gene tree with that of the species tree, rather than rely solely on presence and absence patterns, in order to identify gene birth, transfer, duplication, and loss events. Event penalties were set to hgt: 3, dup: 2, los: 1, and spc: 0. Ultrametric was set to True in order to constrain events temporally. Each run was conducted with 100 gene tree bootstraps in order to increase accuracy (David & Alm, 2011). Individual event timings were defined as the midpoint of the temporal region during which a given event could occur.

## 3 RESULTS

### 3.1 Generation of a species tree and fossil‐calibrated chronogram

**Figure 2:**
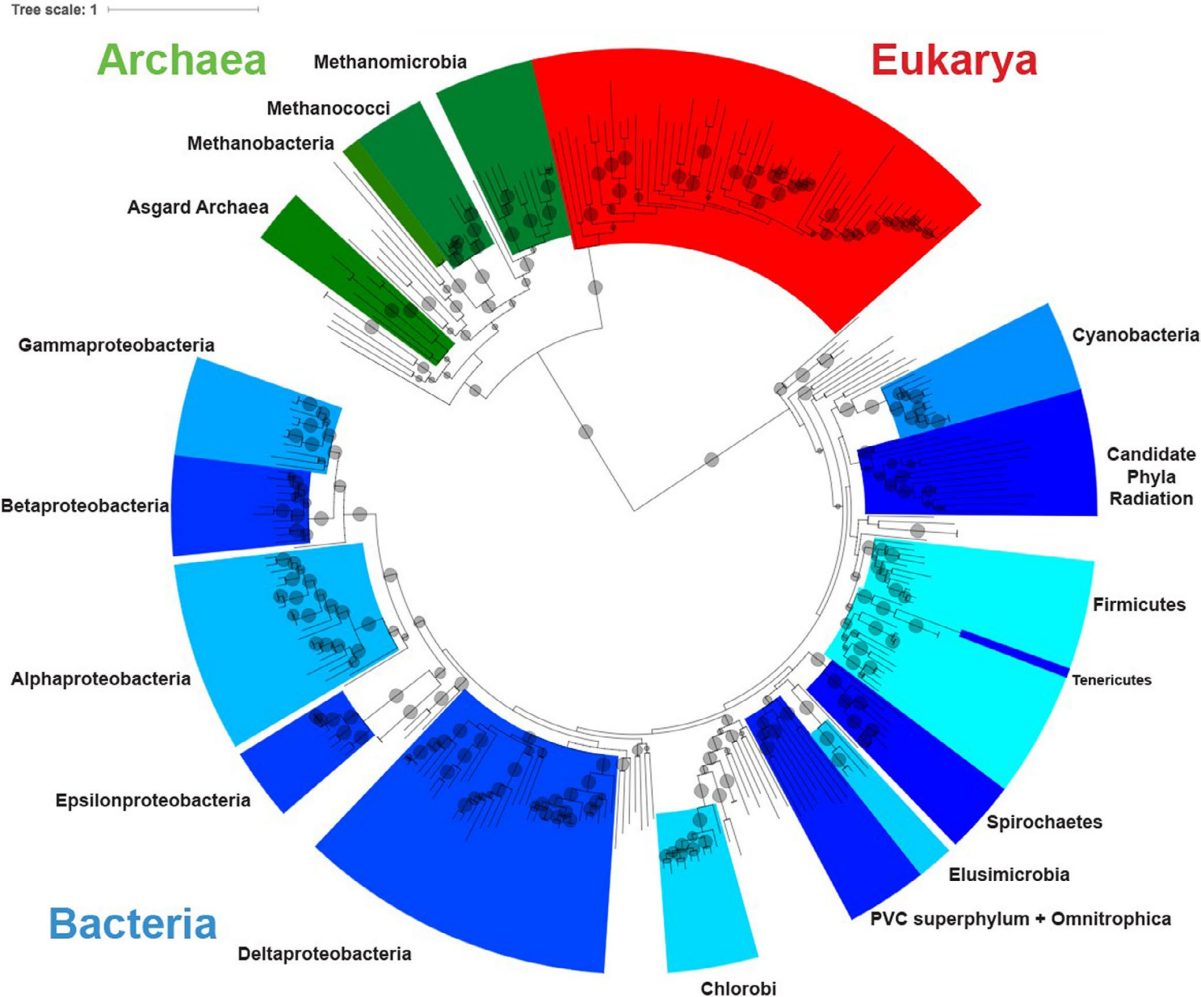
Species tree used for phylogenetic analysis. Maximum likelihood phylogeny based on an alignment of concatenated single‐copy universal proteins from 308 genomes. Well‐represented bacterial and archaeal phyla are labeled in black text. Bacterial clades are labeled in alternating colors of blue, archaea in green, and eukaryotes in red. Bootstrap values (from 100 bootstraps) >50 are shown as transparent gray circles, with larger circles representing higher bootstrap values

We compared all gene trees to a species tree constructed from an alignment of concatenated sequences of 16 single‐copy universal proteins from 308 organisms (Figure 2; genome list deposited to FigShare at https://doi.org/10.6084/m9.figshare.12791747.v1, see below). Monophyly was preserved for most major phyla, with three exceptions: (a) Tenericutes is contained within Firmicutes, (b) the PVC superphylum contains Omnitrophica, and (c) Lentisphaerae is nested within Verrucomicrobia. Additionally, in contrast to a recently published comprehensive tree of life (Hug et al., 2016), our tree does not place the recently discovered Candidate Phyla Radiation (CPR) bacteria as the deepest‐rooted bacterial clade; however, the CPR is placed as a sister group to the Cyanobacteria and Melainabacteria, which is consistent with that phylogeny (Hug et al., 2016). For the purposes of this study, placement of these groups should not greatly affect our results due to the high number of duplication/loss/transfer events we inferred overall across all groups. The species tree used for this analysis is a three‐domain tree, in contrast to recent studies which have shown the addition of the Asgard Archaea to the tree of life to cause Eukaryotes to group within Archaea (Zaremba‐Niedzwiedzka et al., 2017). However, we are agnostic as to the placement of the Asgard superphylum and the eukaryotes on the tree of life, as this was not our focus; the relationship of the three domains to one another should not substantially affect the results shown here due to the relative infrequency of interdomain HGT relative to interdomain HGT, and the exclusion of eukaryotic nitrogen‐cycling genes.

**Figure 3:**
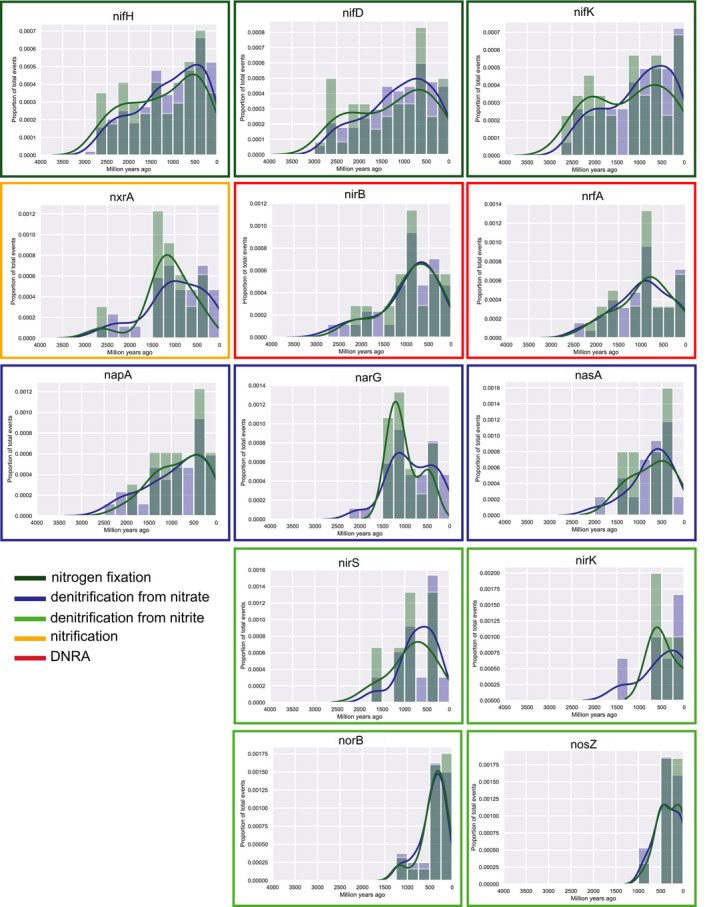
Histograms and density plots of all speciation, loss, duplication, and horizontal gene transfer (HGT) events. All events are shown in blue, HGT events alone are shown in green. Events were inferred by reconciling phylogenetic trees of nitrogen‐cycling genes with a chronogram generated using a CIR clock model and conservative calibration points. Y‐axis represents proportion of all events (blue) or HGT events only (green). Borders around graphs indicate the metabolic process that the gene is involved in. Histograms are ordered according to the earliest time point at which a speciation, loss, duplication, or HGT event occurred. An equivalent figure with dates inferred from the UGAM clock model is shown in Figure S3

We constructed four different chronograms from our species tree using two different clock models (UGAM and CIR) as well as liberal (representing the earliest date for which there is any evidence of a given event based on the current scientific literature) and conservative (representing the earliest date for which there is the most consensus for a given event based on the current scientific literature) fossil calibration points (see Methods). The liberal calibration points yielded an unreasonable root age (>4.5 Ga) and so were not further used for analysis. The UGAM clock model yielded a greater spread in estimated ages for node divergences, with an earlier root age (approximately 4,044.68 ± 143.093 Mya compared to 3,982 ± 131.218 Mya for the CIR clock model). The results shown in Figure 3 and Table 4 derive from the CIR clock model as this has been previously shown to outperform uncorrelated models (Lepage et al., 2007), but the results from the UGAM clock model are shown in Figure S3 and Table S2. All genome lists, alignments, and Newick files have been deposited in FigShare at: https://figshare.com/projects/Radiation_of_nitrogen_cycling_genes_across_the_tree_of_life/87461

### 3.2 Identification of duplication, speciation, loss, and horizontal gene transfer events for nitrogen‐cycling genes

We identified 18 different nitrogen‐cycling genes for analysis of horizontal gene transfer, speciation, duplication, and loss events (Table 2). This methodology is best suited to genes that have many representatives from a diverse suite of taxa. Very few genes from the *hao* and *amo* gene families were identified in these genomes or passed our stringent filtration tests, and thus, they did not yield sufficient data for robust conclusions and were not included in further analyses. We generated maximum likelihood gene trees for each of the remaining genes (see Data Availability Statement) and compared these gene trees to the fossil‐calibrated chronogram in order to identify and infer the timing of birth, duplication, loss, and horizontal gene transfer events for each of these genes. The number of speciation events inferred for each gene in this study was relatively high compared to the number of inferred loss and HGT events (Table 3), with very few duplication events observed. Inferred loss events generally skewed younger than inferred horizontal gene transfer and speciation events. Our results indicate that the iron‐molybdenum nitrogenases (*nifH/nifD/nifK*) were among the oldest genes, originating during the Archean era and beginning to spread across the tree of life via horizontal gene transfer fairly early in Earth history (Figure 3). In contrast, the alternative nitrogenases *anf* and *vnf* were inferred to have arisen and radiated across the tree of life much later (Table 4, Table S2, Figure S4 and S5), but very few *anf* and *vnf* genes were identified among our sample set and, therefore, these results should be treated with caution (Table 2). The denitrifying genes *norB, nosZ, nirK,* and *nirS,* were inferred to have arisen later in Earth history (Table 4) and began to proliferate across the tree of life much more recently, up to approximately 1.5 Ga (Figure 3). These genes encode enzymes that catalyze denitrification processes from nitrite to nitric oxide, nitrous oxide, and dinitrogen gas. The genes *nrfA* and *nirB*, which are involved in the DNRA process, are inferred to have arisen by approximately 2.7–2.2 Ga, and began to increasingly proliferate widely across the tree of life into the Mesoproterozoic. Genes involved in nitrate reduction, including *narG*, *nasA*, and *napA,* were inferred to have arisen relatively early (approximately 2.8 Ga for *narG* and *napA*; approximately 2.3 Ga for *nasA*) (Table 4), and we inferred a few speciation and horizontal gene transfer events for these genes between 2–2.5 Ga, but we did not observe a rise in HGT events for these genes until much later, at approximately 1.5 Ga (Figure 3). We observed a similar trend for *nxrA*, a gene involved in nitrite oxidation, which was inferred to have arisen around 2.8 Ga but did not exhibit a rise in speciation, duplication, or transfer events until approximately 1.5 Ga (Figure 3, Table 4).

**Table 2:** Number of nitrogen‐cycling genes identified within 254 bacterial and archaeal genomes that were included in the analysis. All genes were identified from ORFs using BLASTP with an e‐value cutoff of 10^‐12^, then filtered using the Kofam Koala tool. Nitrogenases were further refined by identifying key residues (see Methods)

Gene	Number of genes identified
*amo*	4
*anfD*	11
*anfK*	10
*hao*	0
*napA*	37
*narG*	34
*nasA*	28
*nifD*	165
*nifK*	92
*nifH*	233
*nirB*	39
*nirK*	10
*nirS*	16
*norB*	36
*nosZ*	21
*nrfA*	48
*nxrA*	34
*vnfD*	6
*vnfK*	6

**Table 3:** Frequencies of events inferred by AnGST for each gene as derived from both the CIR and UGAM clock models

Gene	CIR clock model				UGAM clock model			
	HGT	Speciation	Loss	Duplication	HGT	Speciation	Loss	Duplication
*anfD*	7	7	4	0	7	7	4	0
*anfK*	6	7	4	0	6	7	4	0
*napA*	13	46	23	0	12	50	26	0
*narG*	15	29	15	4	14	32	17	4
*nasA*	5	27	6	1	5	27	6	1
*nifD*	48	176	60	0	51	169	56	0
*nifK*	35	103	48	1	34	107	51	1
*nifH*	68	224	72	12	74	202	56	12
*nirB*	14	34	13	3	13	38	16	3
*nirK*	4	9	5	1	4	9	5	1
*nirS*	7	13	6	1	7	13	6	1
*norB*	26	13	5	2	25	13	5	2
*nosZ*	13	5	0	2	13	5	0	2
*nrfA*	24	34	13	2	23	37	15	2
*nxrA*	13	34	18	4	13	34	18	4
*vnfD*	3	3	1	0	3	3	1	0
*vnfK*	3	3	1	0	3	3	1	0

**Table 4:** Inferred birth dates for nitrogen‐cycling genes based on the chronogram generated using the CIR clock model with conservative calibration points

Gene	Upper node date	95% confidence interval	Lower node date	95% confidence interval	Midpoint between nodes	Geologic era
vnfD	1445.24	1052.37, 1735.83	482.23	210.11, 778.444	963.73	Neoproterozoic
vnfK	1445.24	1052.37, 1735.83	482.23	210.11, 778.444	963.73	Neoproterozoic
nosZ	1325.57	1048.93, 1588.52	1139.65	851.174, 1408.81	1232.61	Mesoproterozoic
nirK	1452.66	1193.07, 1706.53	1343.08	1094.21, 1585.92	1397.87	Mesoproterozoic
nirS	1913.87	1694.13, 2144.31	1423.17	1153.32, 1677.23	1668.52	Paleoproterozoic
norB	1913.87	1694.13, 2144.31	1423.17	1153.32, 1677.23	1668.52	Paleoproterozoic
anfD	2349.58	2153.39, 2583.93	1745.53	1498.39, 1999.54	2047.55	Paleoproterozoic
anfK	2349.58	2153.39, 2583.93	1745.53	1498.39, 1999.54	2047.55	Paleoproterozoic
nasA	2540.50	2351.4, 2778.03	2063.23	1848.31, 2293.91	2301.87	Paleoproterozoic
nrfA	2454.68	2178.51, 2737.37	2267.54	1918.96, 2574.71	2361.11	Paleoproterozoic
nirB	2748.37	2545.88, 2989.62	2603.25	2384.99, 2850.17	2675.81	Neoarchean
nifK	2926.54	2734.92, 3180.11	2629.22	2415.05, 2877.89	2777.88	Neoarchean
nxrA	2816.89	2630.27, 3063.26	2787.46	2603.09, 3029.43	2802.17	Mesoarchean
napA	2816.89	2630.27, 3063.26	2787.46	2603.09, 3029.43	2802.17	Mesoarchean
narG	2816.89	2630.27, 3063.26	2787.46	2603.09, 3029.43	2802.17	Mesoarchean
nifD	2934.72	2744.91, 3187.67	2869.82	2682.33, 3118.74	2902.27	Mesoarchean
nifH	3060.98	2863.64, 3325.02	3026.38	2832.29, 3286.79	3043.68	Mesoarchean

All gene birth events are inferred to occur between nodes on the species chronogram, and, therefore our methods do not allow us to infer specific dates for gene birth events. Here, we list the inferred timing for the earliest possible timing (upper node) and latest possible timing (lower node) with 95% confidence intervals as reported by PhyloBayes, as well as the calculated midpoint between the two node times. Times are reported in millions of years ago. The geological era reported in the right column corresponds to the midpoint between the upper and lower node. Given the uncertainty inherent in the estimation of node timings and inference of events, these are provided for geological reference and should not be taken as definitive timings. The equivalent table, generated using the UGAM clock model, is depicted as Table S2.

Taken together, we observed that genes related to the fixation of nitrogen from dinitrogen gas to ammonium arose early and proliferated across the tree of life relatively quickly, while genes related to nitrate reduction and nitrite oxidation also arose early but did not begin to proliferate across the tree of life until later. Genes related to denitrification, particularly downstream from nitrite, arose much later (Figure 3). Our results regarding the timing of the birth, duplication, speciation, loss and HGT events for specific nitrogen‐cycling genes showed a few differences between the CIR (Figure 3, Table 4) and UGAM clock models (Figure S3, Table S2). However, the overall patterns in the relative timing for the birth and spread of specific genes in the nitrogen cycle were similar regardless of the type of clock models used.

## 4 DISCUSSION

We have focused here on tracking gene birth, duplication, speciation, and particularly horizontal gene transfer events across deep time. The acquisition of new functional genes is particularly important because it can allow clades of microbes to invade new ecological niches. The rate of horizontal gene transfer itself is likely to be related to variables like cell density, co‐localization of donor and recipient, cell diversity, and the types of organisms involved (Gogarten & Townsend, 2005). Although some have argued for a neutral theory of gene transfer in which horizontally acquired genes are not adaptive (Andreani et al., 2017; Gogarten & Townsend, 2005), studies indicating that horizontally transferred genes perform crucial cellular functions and enable adaptation to specific ecological niches suggest that horizontally acquired genes are generally adaptive (Burke et al., 2011; Coleman & Chisholm, 2010; Daubin & Ochman, 2004; McInerney et al., 2017; Moulana et al., 2020; Polz et al., 2013; Popa et al., 2011). Moreover, genes providing selective advantages are more likely to be retained in the genome than genes acquired due to neutral transfer, which are more likely to be purged, thus strengthening the signal of adaptive HGT events in the genomic record. Therefore, any observations of a rise in the relative number of successful horizontal gene transfers for a given gene are likely to give an indication of the relative availability or metabolic importance of a given substrate for that gene. As such, studying the history of HGT of specific genes can provide insights into the points at which possession of such genes provided substantial selective advantages, which can then be used as a metric for when a specific metabolism became feasible or energetically favorable, or when the substrates of specific enzymes became relatively abundant. A crucial caveat to this method, however, is that it cannot infer relative abundance or population sizes of the organisms carrying these genes. Thus, if a specific strain carrying a nitrogen‐metabolizing gene grows in abundance with no transfer of genes to other lineages, our method would observe low rates of transfer for genes involved in that metabolism. Additionally, a poor phylogeny caused by weak phylogenetic signal within the alignment would make it difficult to precisely identify such horizontal gene transfer events. Therefore, these results, while easily overinterpreted, should largely be interpreted in the context of other studies, especially those which use substantially different methodologies.

On the whole, our results provide support for geochemical data indicating that biological nitrogen fixation was an important source of fixed nitrogen in the Archaean (Ossa Ossa et al., 2019; Stüeken et al., 2015). These results also suggest that local sources of nitrate may have been exploited by denitrifying microbes, while denitrifiers using nitrite or downstream products would not have proliferated until much later in Earth history, until the mid‐Proterozoic, well after the Great Oxidation Event. This finding is consistent with previous geochemical studies that documented isotopic evidence of denitrification in the Neoarchean (Garvin et al., 2009b; Godfrey & Falkowski, 2009; Koehler et al., 2019) and Paleoproterozoic (Kipp et al., 2018; Luo et al., 2018; Zerkle, Poulton, et al., 2017). Our results suggest that genes involved in nitrate reduction arose by approximately 2.8 Ga, indicating that nitrate was present and used as a metabolic substrate at that time. However, our data suggest that denitrification may not have been a reliable energy source until about 1.5 Ga and therefore used by a smaller diversity of clades. From the mid‐Proterozoic onwards, nitrate may have been sufficiently bioavailable to make it a more widely used substrate. In the following, we discuss the implications of our results for the evolution of the nitrogen cycle and its relationship to the redox state of the Earth.

### 4.1 Nitrogen fixation

Our data indicate that nitrogen fixation through the use of molybdenum nitrogenase (Nif) is an ancient process, arising by approximately 3.1–2.7 Ga (Table 4). Our results are consistent with previous work from geochemical analyses suggesting that nitrogen fixation must have arisen early in order to support an expanding biosphere (Koehler et al., 2019; Ossa Ossa et al., 2019; Stüeken et al., 2016). In contrast, a previous analysis based on the evolutionary rate of nitrogenase genes suggested that functional Mo‐nitrogenase arose relatively late (approximately 2.2–1.5 Ga) (Boyd, et al., 2011), which may also be consistent with the hypothesis that modern planktonic nitrogen fixers did not become abundant in global oceans until the Neoproterozoic (Sánchez‐Baracaldo et al., 2014). Our results, which are based on reconciliation of gene trees with a chronogram inferred from universally conserved, single‐copy genes, are instead consistent with recent nitrogen isotope evidence for biological nitrogen fixation back to at least 3.2 Ga (Stüeken et al., 2015). It is also consistent with phylogenetics work suggesting that molybdenum nitrogenase arose early in the evolution of life on Earth (Raymond et al., 2004). Similarly, phylogenetic reconstructions showing that Mo‐nitrogenases arose before V‐ and Fe‐nitrogenases are consistent with this conclusion (Garcia et al., 2020). It has been argued that nitrogenase was present in LUCA (Weiss et al., 2016), though that has been disputed (Berkemer & McGlynn, 2020; Boyd, et al., 2011; Mus et al., 2019) and our results do not resolve this issue. However, the identification of horizontal gene transfer events for nitrogenase subunits during the Archean (Figure 3) suggest that this metabolism may have been abundant and beneficial enough during this time period to have been successfully transferred and retained in microbial genomes.

Our results support the argument that abiotic sources of fixed nitrogen were unlikely to be significant enough to sustain the early biosphere (Canfield et al., 2010; Raymond et al., 2004). It has been proposed that early life received its nitrogen from the lightning‐catalyzed reaction between N_2_ and CO_2_ as a source of NO_x_, and that a steady reduction in atmospheric CO_2_ levels reduced this flux, eventually leading to a nitrogen crisis around 2.2 Ga that would have favored the rise and spread of biological nitrogen fixation (Navarro‐González et al., 2001). In contrast, several studies have concluded that abiotic nitrogen fixation on the early Earth would produce 50‐ to 5000‐fold lower rates of fixed nitrogen than what is contained in the modern ocean, making nitrogen highly limiting to the early biosphere, even at higher CO_2_ levels (Canfield et al., 2010). Our data suggest that biological nitrogen fixation arose and proliferated early and that acquisition of the nitrogenase enzyme provided enough of a selective advantage to be successfully transferred across lineages during the early Archaean, supporting the notion that fixed nitrogen was not widely available.

Moreover, if the radiation of biological N_2_ uptake dates back to the early Archean, it may have had a significant impact on atmospheric pressure. Today, nitrogen makes up approximately 78% of the Earth's modern atmosphere by volume, predominantly as N_2_ gas. The atmosphere of the early Earth was probably also N_2_‐rich, but while some models suggest a 2–4 times larger atmospheric N_2_ reservoir in the Archean (Johnson & Goldblatt, 2018), proxy evidence ranges from near modern values (Avice et al., 2018; Marty et al., 2013) to less than half of today's reservoir (Som et al., 2016). If atmospheric N_2_ pressure changed over time, it is likely that biological activity would have played a major role in driving these changes by enhancing nitrogen burial in sediments and by accelerating oxidative weathering of nitrogen from continental crust (Stüeken, et al., 2016; Zerkle & Mikhail, 2017). If nitrogen was fixed by nitrogenase early in the Archaean, as indicated by our results, then biological nitrogen burial began long before the onset of oxidative weathering in the Neoarchean (Stüeken et al., 2012) which makes it possible that atmospheric N_2_ decreased over the course of the Archean. Although further paleobarometric proxies are needed to verify this trend, our results provide an important anchor point for the onset of biological N_2_ drawdown.

Lastly, though we identified very few iron and vanadium nitrogenases (*anf* and *vnf*, respectively) in our datasets, our results tentatively suggest that these nitrogenases radiated across the tree of life later than the iron‐molybdenum nitrogenases (*nif*), which supports conclusions from other phylogenetics studies (Garcia et al., 2020). The early rise of Mo‐dependent nitrogenase would have required a source of Mo to act as a cofactor for Mo‐containing nitrogenases, implying that nanomolar levels of dissolved Mo (Reinhard et al., 2013; Scott et al., 2008), presumably derived from anoxic weathering or hydrothermal sources of molybdenum to the early ocean, were sufficient for the development of Nif and the proliferation of nitrogen fixers. Oxidative weathering was therefore evidently not required for the onset of biological nitrogen fixation (cf., Boyd, et al., 2011). We speculate that the evolutionary pressure for the diversification of alternative nitrogenases arose in the Neoproterozoic with the rise of eukaryotic algae (Brocks et al., 2017), which may have substantially increased the nitrogen demand in the global ocean. Vanadium and iron‐based nitrogenases are less efficient at fixing nitrogen than Nif (Mus et al., 2018), which perhaps lowers the probability of successful gene transfer for Vnf and Anf. However, when nitrogen demand increased in the environment, it is conceivable that more of these genes were shared successfully. Another hypothesis to explain our data is that the gene transfer of alternative nitrogenases was affected by global climate. Vnf becomes more efficient than Nif at cold temperatures (Miller & Eady, 1988) and the activity of Nif‐using cyanobacteria decreases at high latitudes (Brauer et al., 2013). Thus, the cold climate of the Cryogenian’Snowball Earth’ period (720–635 Ma) (Hoffman et al., 2017) may have increased the fitness of organisms possessing Vnf. However, these hypotheses remain speculative until more genomic data for Vnf and Anf has been acquired. In any case, it is important to keep in mind that the origin of alternative nitrogenases likely occurred long before the Neoproterozoic. Vanadium nitrogenase in *Azotobacter vinelandii* has been shown to reduce carbon monoxide (CO) to the hydrocarbons ethylene (C_2_H_2_), ethane (C_2_H_6_), and propane (C_3_H_8_) (Lee et al., 2010), which supports the origin of V‐nitrogenase in the Archean eon when CO was more abundant in the atmosphere (Anbar & Knoll, 2002). Additionally, V was bioavailable in Archean ocean waters under slightly acidic conditions (Moore et al., 2020). The genomes of more V‐nitrogenase and Fe‐nitrogenase organisms must be sequenced to better understand the evolutionary history of these alternative nitrogenases.

### 4.2 Nitrogen cycling prior to the oxygenation of the oceans and atmosphere

Although our results suggest that nitrogen fixation was an important process on the early Earth, microbial metabolisms making use of more oxidized forms of nitrogen, particularly through the process of denitrification downstream from nitrite, do not appear to have arisen and spread across the tree of life until much later. Some early steps in the nitrogen cycle, such as nitrate reduction to nitrite via *nasA*, *narG,* or *napA*, appear to have arisen relatively early (in the late Archean or early Proterozoic), consistent with geochemical record of denitrification at that time (Godfrey & Falkowski, 2009; Koehler et al., 2019). However, we did not identify many horizontal gene transfer events early in the evolutionary history of these genes, and their proliferation across the tree of life only began in earnest much later, approximately 1.5 Ga. Our results therefore suggest that nitrate availability was restricted in space and/or time. Indeed, thermodynamic constraints and environmentally resolved geochemical datasets show that the anoxic deep ocean of the Precambrian contained ammonium while nitrate was restricted to surface waters (Stüeken et al., 2016; Yang et al., 2019). It is therefore likely that the nitrate‐reducing genes appeared in locally oxygenated regions of the Archean surface ocean, which have been inferred from isotopic studies back to 3.0 Ga (Olson et al., 2013; Planavsky et al., 2014). In such oxygen oases, oxygen concentrations may have reached micromolar concentrations (Olson et al., 2013), which is high enough for the complete oxidation of ammonium to nitrite and nitrate (Lipschultz et al., 1990). Alternatively, a small flux of nitrogen oxides from lightning and volcanism may have existed (Mather et al., 2004; Navarro‐González et al., 2001) and maintained populations of nitrate‐reducing denitrifiers. Denitrification is a highly energy‐yielding metabolism (Schoepp‐Cothenet et al., 2013), and therefore even a small source flux of nitrate to the early ocean would probably have been exploited. However, we stress that denitrification left its geochemical mark in only a few Archean basins (Garvin et al., 2009b; Godfrey & Falkowski, 2009; Koehler et al., 2019), while other localities show no geochemical evidence of nitrate reduction (Ossa Ossa et al., 2019). This observation may argue against a significant contribution of lightning‐derived nitrogen oxides, which we would expect to have been more uniformly distributed.

Our results suggest that genes for nitrite‐metabolizing enzymes were not frequently transferred during the early Archean, which may imply that nitrite‐metabolizing genes were not widespread in Archean ecosystems. It is possible that only specific taxa were responsible for the conversion of nitrite into nitrate or ammonium. Alternatively, the conversion of nitrite to ammonium may have happened abiotically. Ferrous iron (Fe^2+^), which is thought to have been abundant in the early anoxic ocean, may have readily reduced nitrite to ammonium or nitrogen gas without biological intervention (Canfield et al., 2010; Summers & Chang, 1993). Nitrite levels during this time period were therefore likely too low to cause substantial proliferation of genes for enzymes which take nitrite as a substrate, while nitrate and ammonium concentration were high enough for selection to favor biological metabolism of these molecules by diverse microorganisms.

### 4.3 Effects of increased oxygen levels on the nitrogen cycle

Multiple lines of evidence point to increasing oxygenation of surface environments from 2.75 Ga onwards, culminating in the Great Oxidation Event (GOE) at 2.3 Ga (Crowe et al., 2013; Lyons et al., 2014; Noffke et al., 2007; Planavsky et al., 2014). Our data suggest that the genes *nrfA* and *nirB,* which reduce a variety of oxidized forms of nitrogen to ammonium through the DNRA pathway, arose in the Neoarchean or Paleoproterozoic, but the relative number of HGT events for this gene began to rise approximately around the time of the GOE. Unlike denitrification to N_2_ gas, DNRA retains the reduced nitrogen product in the system as aqueous NH_4_^+^, which may have prevented a potential ‘nitrogen crisis’ during the wake of the GOE (Falkowski & Godfrey, 2008). The isotopic fingerprint of DNRA can so far not be distinguished from denitrification in the geochemical record, and thus, our results provide the first indication that this metabolism may have been of greater ecological importance than previously proposed. DNRA rather than denitrification may therefore explain geochemical evidence of biological nitrate reduction in the Neoarchean and early Proterozoic (Garvin et al., 2009b; Godfrey & Falkowski, 2009; Kipp et al., 2018; Zerkle et al., 2017), that is, long before our observed radiation in denitrification genes. This observation may suggest that these early nitrate‐reducing ecosystems were primarily performing DNRA, consistent with iron‐rich conditions in the Archean and Paleoproterozoic ocean (Michiels et al., 2017).

After approximately 1.5 Ga, the relative number of HGT events for enzymes involved in the modern denitrification pathway began to rise. Additionally, our data suggest that the gene *nxr*, which oxidizes nitrite into nitrate, arose relatively early (approximately 2.8 Ga) but did not begin to spread across the tree of life until ~1.5 Ga. One possibility is that increased copper availability facilitated the spread of nitrification/denitrification, which require enzymes that rely on copper cofactors (Moore et al., 2017). However, geochemical evidence of increasing copper concentrations in seawater at 1.5 Ga is so far lacking. Another possibility is that the expansion of these metabolisms is linked to a Mesoproterozoic rise in oxygen, which has recently been proposed in several geochemical studies (Canfield et al., 2018; Cox et al., 2016; Shang et al., 2019; Zhang et al., 2018). Higher oxygen levels would have led to an expansion of the marine nitrate reservoir, which may in turn have been critical for the rise of eukaryotes around this time (Anbar & Knoll, 2002; Knoll & Nowak, 2017).

The primary substrate for many of the enzymes that demonstrated this late rise in the number of HGT events is nitrite, suggesting that nitrite levels may have increased at this time, such that selection favored the spread of nitrite‐metabolizing enzymes across the tree of life. Nitrite is thermodynamically unstable in oxic conditions, but it occurs transiently in chemoclines in the modern ocean because it is abundantly produced as an intermediate in nitrification and denitrification pathways (Wada & Hatton, 1971). A more vigorous aerobic nitrogen cycle in the Proterozoic may thus be linked to the establishment of a more permanent dynamic nitrite reservoir. Abiotic reduction of nitrite by Fe^2+^, which was perhaps dominant during the Archean (see above), may have slowed down with the deepening of the chemocline after the GOE.

Several caveats must be kept in mind while interpreting these results. As mentioned above, the accuracy of molecular clocks is constrained by the models used to infer dates and the accuracy of the fossil‐based time points used for clock calibration. We sought to minimize the influence of these parameters by using both liberal and conservative time points and by employing two different clock models, but nevertheless these limitations must be taken into consideration. Thus, we emphasize the relative rather than the absolute timing of the events identified here. Importantly, the results generated through the use of liberal calibration points are not intended to be taken as an older bound for these gene proliferations, but rather as a test of the sensitivity of our results to the specifications of the molecular clock. Moreover, the methods used to identify genes used in the analysis were designed to be conservative so as to reduce the possibility that spurious genes were included in the analysis, but these methods are inherently limited by the quality of the databases used for annotation. These conservative methods precluded the inclusion of *hao* and *amo,* which are involved in the oxidation of ammonia to nitrite and nitrogen gas, in our analysis. Others have observed that archaeal ammonia oxidizers most likely originated during the GOE and began to spread through the shallow ocean approximately 800 Mya (Ren et al., 2019). As new genes and genomes continue to be sequenced and analyzed for gene function, the quality of such analyses will improve over time.

## 5 CONCLUSION

Our results show that biological nitrogen fixation appears to have arisen and proliferated early in the Archaean, and that genes in the denitrification pathway and genes related to the consumption of nitrite and its downstream products began to proliferate across the tree of life following the oxygenation of Earth's atmosphere and oceans. Moreover, our results support the hypothesis that the molybdenum‐based variety of nitrogenase diversified much earlier than alternative nitrogenases, which may only have become important with the increasing nitrogen demand of algae in the Neoproterozoic, implying that molybdenum was not a limiting resource in the Archean ocean. Furthermore, our data provide the first indirect evidence for a small source of nitrate to the early ocean, possibly as a result of lightning and volcanism or from localized oxygen oases. Some of this nitrate appears to have been used for nitrate reduction to ammonium (DNRA), which may have helped overcome nitrogen limitation by retaining fixed nitrogen in the system. DNRA rather than denitrification may explain some of the isotopic records of biological nitrate utilization in the Archean and early Proterozoic. We cannot confirm the hypothesized suppression of N_2_O‐metabolizing enzymes in the mid‐Proterozoic, but our results are consistent with vigorous nitrification and denitrification after the GOE, in particular from 1.5 Ga onwards, which may have led to leakage of N_2_O into the atmosphere and the stabilization of global climate. The proliferation of key nitrogen‐metabolizing genes across the tree of life at different points in Earth's redox history provides important insights into the evolution and radiation of microbial metabolisms on the early Earth in response to major environmental transitions and supports the notion that increasing nitrate levels in the mid‐Proterozoic may have contributed to the rise of eukaryotic life.

## Supporting information

Fig S1‐S5

Table S1‐S2

## Data Availability

The data that support the findings of this study are openly available in FigShare at the following DOIs: https://doi.org/10.6084/m9.figshare.12791747.v1 (list of genomes), https://doi.org/10.6084/m9.figshare.12791750.v1 (UGAM chronogram), https://doi.org/10.6084/m9.figshare.12791756.v1 (CIR chronogram), https://doi.org/10.6084/m9.figshare.12791759.v1 (gene alignments), https://doi.org/10.6084/m9.figshare.12791765.v1 (gene trees), https://doi.org/10.6084/m9.figshare.12791966.v1 (species tree and alignment).
